# Breeding habitat and nest‐site selection by an obligatory “nest‐cleptoparasite”, the Amur Falcon *Falco amurensis*

**DOI:** 10.1002/ece3.5878

**Published:** 2019-12-02

**Authors:** Martin Frommhold, Arend Heim, Mikhail Barabanov, Franziska Maier, Ralf‐Udo Mühle, Sergei M. Smirenski, Wieland Heim

**Affiliations:** ^1^ Institute for Environmental Sciences and Geography University of Potsdam Potsdam‐Golm Germany; ^2^ Institute of Geography University of Leipzig Leipzig Germany; ^3^ Institute of Biochemistry and Biology University of Potsdam Potsdam Germany; ^4^ Landratsamt Neustadt an der Waldnaab Germany; ^5^ Ecological Field Station Guelpe University of Potsdam Havelaue Germany; ^6^ Muraviovka Park for Sustainable Land Use Blagoveshchensk Russia; ^7^ Institute of Landscape Ecology University of Muenster Muenster Germany

**Keywords:** cleptoparasitism, fire, habitat use, machine learning, magpie, nest‐site selection, random forest

## Abstract

The selection of a nest site is crucial for successful reproduction of birds. Animals which re‐use or occupy nest sites constructed by other species often have limited choice. Little is known about the criteria of nest‐stealing species to choose suitable nesting sites and habitats. Here, we analyze breeding‐site selection of an obligatory “nest‐cleptoparasite”, the Amur Falcon *Falco amurensis*. We collected data on nest sites at Muraviovka Park in the Russian Far East, where the species breeds exclusively in nests of the Eurasian Magpie *Pica pica*. We sampled 117 Eurasian Magpie nests, 38 of which were occupied by Amur Falcons. Nest‐specific variables were assessed, and a recently developed habitat classification map was used to derive landscape metrics. We found that Amur Falcons chose a wide range of nesting sites, but significantly preferred nests with a domed roof. Breeding pairs of Eurasian Hobby *Falco subbuteo* and Eurasian Magpie were often found to breed near the nest in about the same distance as neighboring Amur Falcon pairs. Additionally, the occurrence of the species was positively associated with bare soil cover, forest cover, and shrub patches within their home range and negatively with the distance to wetlands. Areas of wetlands and fallow land might be used for foraging since Amur Falcons mostly depend on an insect diet. Additionally, we found that rarely burned habitats were preferred. Overall, the effect of landscape variables on the choice of actual nest sites appeared to be rather small. We used different classification methods to predict the probability of occurrence, of which the *Random forest* method showed the highest accuracy. The areas determined as suitable habitat showed a high concordance with the actual nest locations. We conclude that Amur Falcons prefer to occupy newly built (domed) nests to ensure high nest quality, as well as nests surrounded by available feeding habitats.

## Graphical abstract

We analyzed the breeding‐site selection of an obligatory nest‐cleptoparasite, the Amur Falcon (*Falco amurensis*). Based on nest site characteristics sampled in the field and a digital habitat map we demonstrate that Amur Falcons select specifically nests newly built by Magpies (*Pica pica*) which are surrounded by favourable feeding habitat.

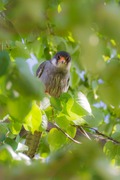

## 1 INTRODUCTION

The selection of a proper breeding site is a key factor for future breeding success. The actual choice of a breeding site is a dynamic process shaped by morphological, physiological, and behavioral adaptations (Wiens, 1992; Winkler, 1988). On a landscape scale, structural elements such as habitat heterogeneity and vegetation type play a role (Newton, 1979). Microhabitat variables regarding the nest and its immediate vicinity are of importance as well, such as vegetation structure, the thermal environment of the nest, or factors offering concealment (Cody, 1985).

Not all species built their own nest, but the usurpation of nests has gained little attention, although it occurs among many species (Lindell, 1996). “Nest‐cleptoparasitism” can be defined as one form of stealing spatial resources such as nest sites and is far from being limited to birds. For example, bumblebee species are known to steal nests from cavity‐nesting birds (Jablonski, Cho, Song, & Kang, 2013). Flying squirrels *Glaucomys* spec. and Red‐bellied Woodpeckers *Melanerpes carolinus* commonly steal nests from the hole‐producing Red‐cockaded Woodpecker *Picoides borealis* (Kappes, 1997; Mazgajski, 2013). But this phenomenon is not limited to cavity‐nesting birds: Matsui, Hisaka, and Takagi (2010) found that Ship Rats *Rattus rattus* usurp open‐cup bird nests for roosting or breeding. In birds, nest stealing was observed in numerous species out of 17 families (Lindell, 1996). Cavities and enclosed nests are much more likely to be usurped than open‐cup nests (Lindell, 1996), and nest usurpation is more common in moderately open habitats with limited structural heterogeneity in the vegetation (Doherty & Grubb, 2002; Lindell, 1996). Little is known about how “nest‐cleptoparasites” choose a nest, and whether features of the nest itself or the surrounding habitat are more important.

The Amur Falcon is a perfect model species to study the site selection of a “nest‐cleptoparasite,” since it is known to exclusively occupy nests built by Eurasian Magpies (hereafter referred to as “magpie”), and the nests are easy to find (Leader, 2001; Zhou, Wang, Liu, Lei, & Gao, 2009). “Nest‐cleptoparasitism” might have evolved in this species because of late arrival on the breeding grounds caused by an exceptional long transoceanic spring migration route from their South‐African wintering areas back to their breeding sites in East Asia (Kumar, 2014; Zhou et al., 2009). Concerning habitat preferences, it is known that Amur Falcons favor open areas, wetlands, and forest edges (Brazil, 2009). Land use intensification and climate change may restrict potential habitats and numbers of this species (Pietersen & Symes, 2010; Symes & Woodborne, 2010). Man‐made fires occur on a regular basis in the floodplain of the Amur river, and those events probably act as a limiting factor for breeding birds in the study area (Heim et al., 2019). Since Amur Falcons depend on the availability of nests built by other birds, it remains unclear, if there are further aspects of the environment such as landscape structure or characteristics of the nest itself that influence the choice of their breeding site (Zhou et al., 2009).

Machine learning algorithms are state‐of‐the‐art statistics that allow for a classification and prediction of cases where many variables and fewer observations are available. Particularly, random forest is described as a very accurate tool to detect influential variables compared to other classification methods. Multiple predictive models are created (within one run) and aggregated to improve the accuracy of prediction. A further benefit is the implied variable importance measure (Kabacoff, 2015).

With this study, we aim to (a) identify predictor variables on local and landscape scale for the nest‐site selection of the Amur Falcon and (b) apply machine learning algorithms to test which classification method is most accurate in predicting the nests occupied by Amur Falcons.

## 2 METHODS

### 2.1 Study area

**Figure 1:**
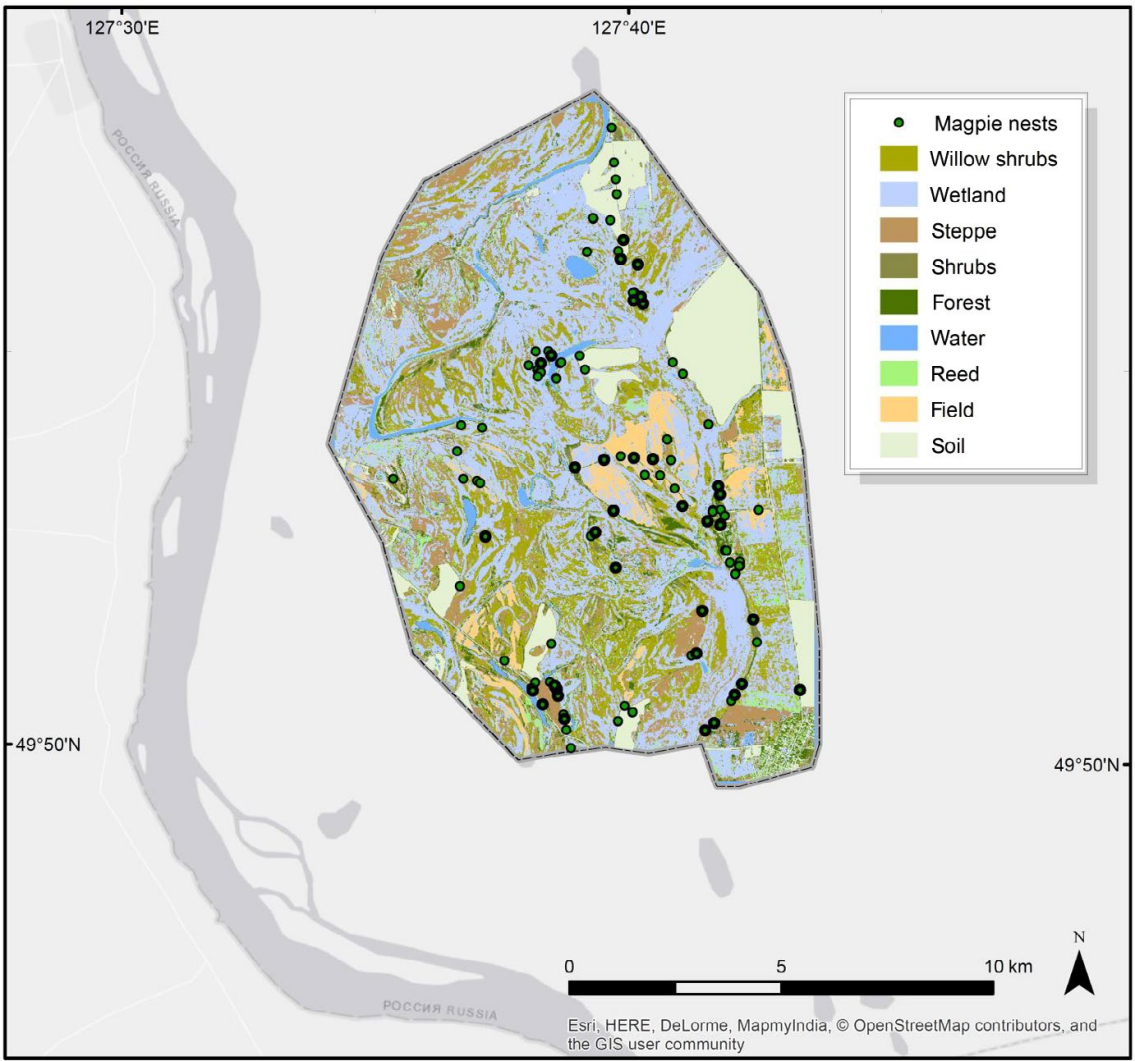
The study area, containing the located magpie nests (*n* = 117) and the classified habitat types (Heim, 2018). The darker circles represent the nests occupied by Amur Falcons

Our study area, the Muraviovka Park for Sustainable Land Use and its surroundings, is situated at the southern end of the Zeya‐Bureya plain on the middle section of the Amur River in the Russian Far East (Figure 1). The area stretches about 16 km from south to north and 11.5 km from east to west, covering an area of about 13,289 ha. The valley of the Amur River and its first terraces ranges in altitudes from 105 to 348 m above sea level.

The landscape is dominated by wetlands (6 346 ha) with *Carex meyeriana*, *Carex lasiocarpa*, *Iris laevigata*, and *Menyanthes trifoliata* (Akhtymaov, Morozova, & Boldovski, 2002). Other land cover types include agricultural fields (480 ha) with changing crops from year to year such as soy and buckwheat and fallow fields (1,392 ha). The forest islands (475 ha) contain species of *Quercus mongolica*, *Betula dahurica*, and *Lespedeza bicolor*; reed (323 ha) with a dominant vegetation of *Phragmites australis*; and shrubs (268 ha) comprising *Corylus heterophylla*, *Salix bebbiana*, and *Lespedeza bicolor* (Akhtymaov et al., 2002).

One year after its establishment in 1994, the Park and its adjacent territories became part of the Ramsar List of Wetlands of International Importance. The Amur Bird Project has been investigating the threatened avifauna together with the staff of Muraviovka Park since 2011 (Heim & Smirenski, 2013, 2017).

### 2.2 Data collection

**Figure 2:**
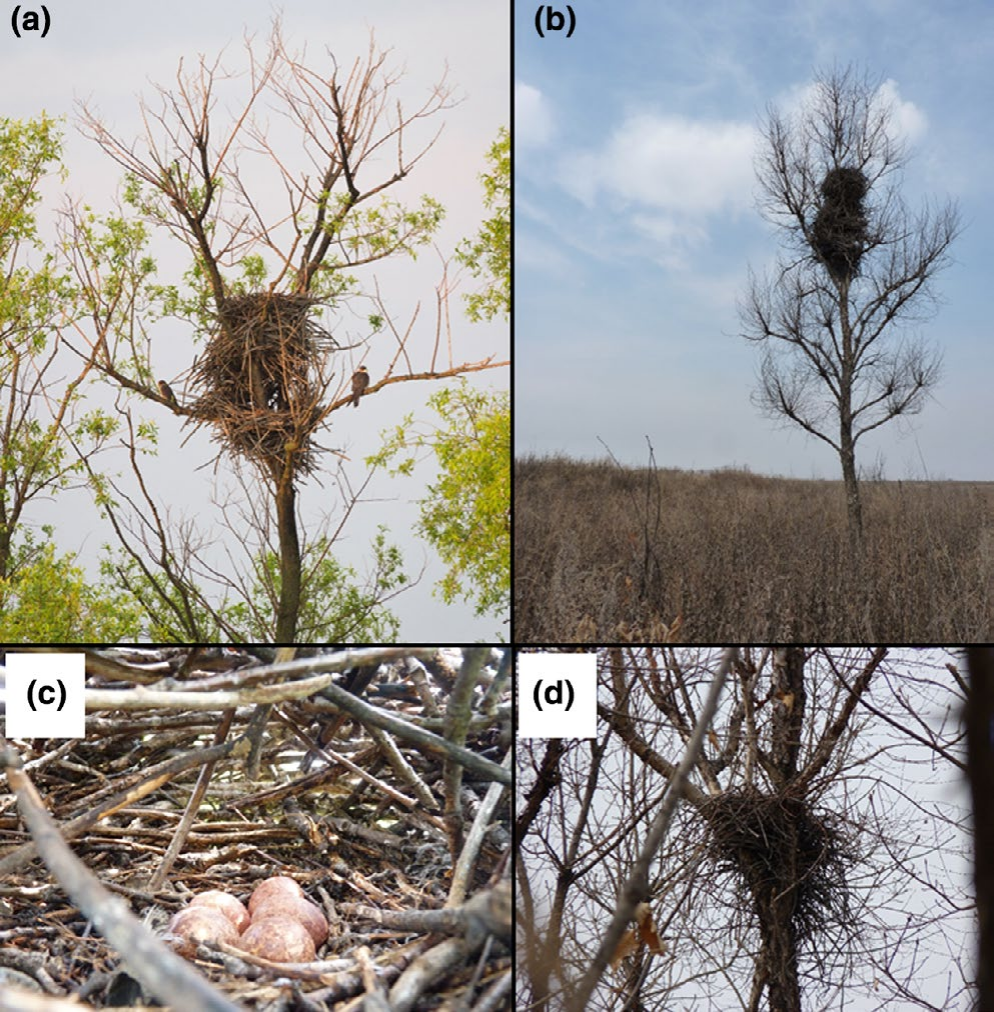
(a) Eurasian Magpie nest occupied by Amur Falcons (A. Heim), (b) Several magpie nests in one tree, where Amur Falcon and Eurasian Magpie bred together (W. Heim), (c) Amur Falcon clutch in a roofed nest (W. Heim), (d) Old nest without roof, with a Long‐eared Owl breeding on top (W. Heim)

We searched the complete study area for nests of magpies, other corvids, and raptors during April–July 2013. Nests were easily located due to the limited number of trees in the area (Figure 2). We collected data on their location using a handheld GPS (Garmin eTrex 10) and assessed the following nest‐specific variables: tree genera, nest height, status of the roof (old magpie nests often lose their roof), and nest content (breeding species, number of eggs or chicks). For the latter variable, the trees were climbed or a prolonged stick with an integrated camera was used to correctly identify the status of the nest.

### 2.3 Data analysis

Data were checked for consistency, and nest locations were intersected with the habitat classification map (Heim, 2018) using *ArcGIS* (version 10.4). Around each nest location, a buffer with a radius of 2,500 m was created that approximately represents the home range of the individual breeding pair. The decision about the size of the home range (1,963.5 ha) was based on references regarding home range estimations for the closely related Red‐footed Falcon *Falco vespertinus* (38–3,467 ha; Fehérvári, Harnos, Neidert, Solt, & Palatitz, 2009; Palatitz, Solt, Horváth, & Kotymán, 2015). Subsequently, buffers were intersected with the habitat classification map to obtain the particular set of habitat patches around each nest. With the help of a fire frequency map (Heim et al., 2019), we tested for an influence of fire on the species occurrence.

The created surroundings of the nests were used for a further calculation of different landscape metrics using the software *Fragstats* (version 4.2.1). The environmental variables on a landscape level included proximity, area‐edge, shape, aggregation, and diversity metrics. The splitting index refers to the fragmentation of the landscape (McGarigal, 2017; Schindler, Poirazidis, & Wrbka, 2013). High values represent a mosaic‐like structure with a higher diversity of different habitat patches. The landscape metric perimeter–area fractal dimension expresses the complexity of the perimeter–area ratio and accounts for the rise or decrease of environmental gradients between patches of a landscape (McGarigal, 2017; Schindler, von Wehrden, Poirazidis, Wrbka, & Kati, 2008; Wang & Malanson, 2007).

Descriptive statistics were carried out and distributions were tested for the following statistical applications with the statistical software *R* (version 3.3.3). In order to start the statistical analyses, the categorical variables such as roof (unknown, no, yes), nesting habitat (willow shrubs, wetland, steppe, shrubs, forest, water, reed, field, bare soil), and tree genera (*Betula*, *Quercus*, *Prunus*, *Ulmus*, *Salix*, *Populus*, *Tilia*, *Crataegus*, dead unidentified tree) were transformed into factor variables.

Results accounting for the differences among the nest occupants are presented by using the median and the median absolute deviation (MAD). The MAD is an alternative to the standard deviation or the interquartile range and considered as a robust scale measure, especially in the presence of outliers. It is calculated by finding the median of absolute deviations from the median (Leys, Ley, Klein, Bernard, & Licata, 2013; Rousseeuw & Croux, 1993).

The dataset was divided into a training (70%), test (15%), and validation (15%) set. With the help of the R package *Rattle* (Williams, 2009), the classification methods *decision trees* and *random forest* were tested. Decision trees are built by the creation of binary splits of the training data on every predictor variable, and the structure of the algorithm allows classifying every new observation into one of two groups. The aim is to construct most homogeneous subsets of the data. The classification threshold can be taken from the pictorial graph of the decision tree (Kabacoff, 2015). Random forest combines many classification trees to produce more accurate classifications (Cutler et al., 2007).

**Figure 3:**
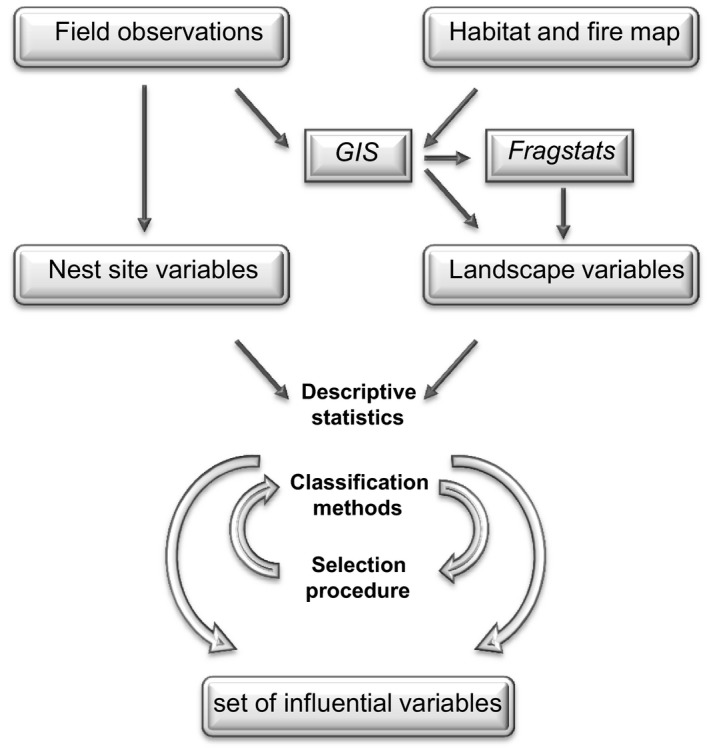
Scheme of the workflow used in the analysis on the nest‐site selection of Amur Falcons

First, all variables were incorporated into the machine learning algorithms. The splitting variables of the *decision trees*, the variable importance measure of the *random forest* application, and the *p*‐values of the *chi‐square* (*χ* ^2^) test from the *logistic regression* served as indicators for influential variables. Decision trees as well as random forests overestimate variables with many categories. Those variables are divided in many auxiliary variables and therefore are more likely to be chosen. Due to this bias toward variables with many classes, nesting habitat and tree genera had to be excluded. Minimum buckets in the *classification trees* were put to seven, and the numbers of trees for the *random forest* application were manually changed to 5,000 to obtain better statistical results. The model run started with 63 variables and subsequent underperformers were removed. Depending on the lowest Akaike information criterion (AIC) values, a set of variables was chosen and incorporated into the classification procedure (Figure 3).

An implied variable importance measure of the random forest application is called Mean Decrease Accuracy (MDA). The MDA shows the decrease in accuracy of the model performance by an error rate calculated with and without the variable. The error rate is calculated for every predictor and then averaged over all constructed trees, which used this specific variable. Predictors with high MDA values are seen as important in the classification of the data, as the predictive accuracy of the model would decrease, if those variables would be left out during calculation (Breiman, 2001).

The performances of the models, each of which includes different sets of predictor variables, were compared using the area under the curve (AUC) values and the overall error. An AUC value of 1 resembles a perfect fit, and the overall error validates the accuracy of the classification algorithms by accounting for all the misclassified cases (Williams, 2009).

## 3 RESULTS

### 3.1 Descriptive statistics

A total of 117 nests were found, all of them built by Eurasian Magpies. Thirty‐eight of those were occupied by Amur Falcons (32.0%), Twenty‐six by Eurasian Magpies (22.0%), six by Northern Long‐eared Owls *Asio otus* (5.0%), four by Siberian Chipmunks *Eutamias sibiricus* (3.0%), and one each by a Daurian Jackdaw *Corvus dauuricus* and a Eurasian Hobby *Falco subbuteo* (~1.0% each). Forty‐one nests were found to be empty (35.0%).

**Figure 4:**
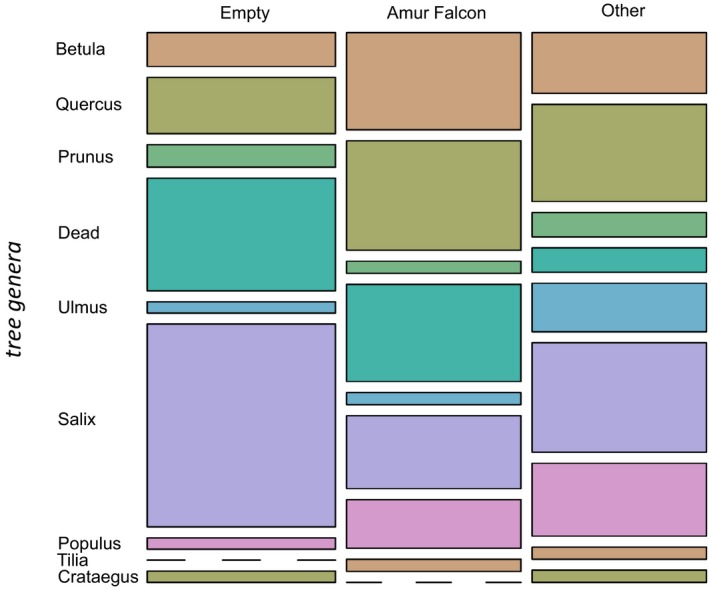
Mosaic plot of the tree genera sorted by the groups empty nests (empty), Amur Falcons, and other species (other). Each sum of a group represents 100%

Recorded nesting trees (all species) were *Salix* spp. (28.0%), *Quercus* spp. (19.0%), unidentified dead trees (17.0%), *Betula* spp. (14.0%), *Populus* spp. (9.0%), *Ulmus* spp. (5.0%), *Prunus* spp. (4.0%), *Tilia* spp. (2.0%), and *Crataegus* spp. (2.0%). Amur Falcons were frequently occupying a nest in *Quercus* spp. (23.7%), *Betula* spp., and dead trees (both accounting for 21.1%). *Salix* spp. (15.8%), *Populus* spp. (10.5%), *Prunus* spp, *Ulmus* spp., and *Tilia* spp. (for all three 2.6%) were rarely chosen as a nest‐site tree for this species. Compared to Amur Falcons, other species picked dead trees less often (5.3%; Figure 4).

The landscape in the study area was dominated by wetland habitats accounting for 47.8%. Wetland patches contributed to 49.4 ± 4.2% of the overall habitat composition within the presumed home ranges of Amur Falcons, whereas shrubs displayed the smallest proportion of about 1.9 ± 0.3%. Amur Falcon home ranges showed the largest proportion of forest patches of 5.07 ± 0.56% in contrast to the groups of other species and empty nests. The greatest amount of bare soil patches can be assigned to the home ranges of Amur Falcons (7.7 ± 2.9%). In comparison between the habitat types, the proportion of forests and shrubs within the study area was low (3.6% and 2%, respectively). However, more than half of the magpie nests were located there (56.4%).

**Figure 5:**
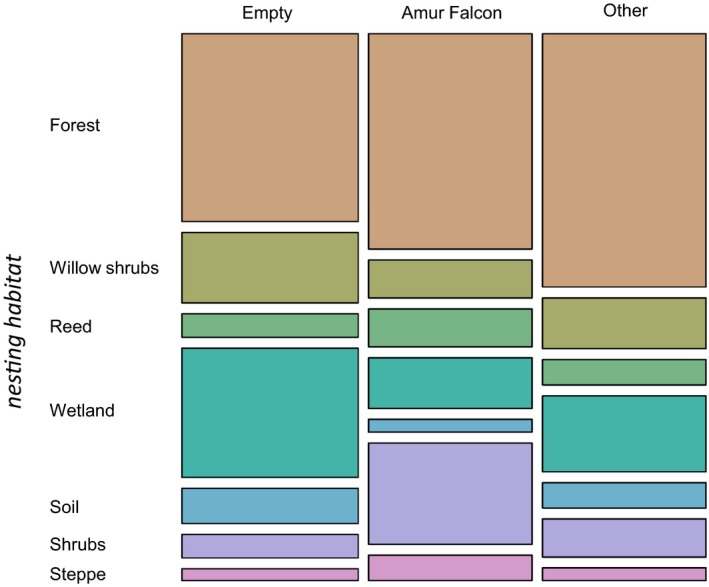
Mosaic plot of nesting habitat sorted by the groups of empty nests (empty), Amur Falcons, and other species (other)

The following differences were identified by analyzing the habitat types and the abundance of nesting sites in each of them (Figure 5). The group of other species was most commonly found in forest patches (52.6%), followed by Amur Falcons (44.7%) and empty nests (42.1%). Empty nests were found most frequently within wetlands (29%) in comparison with the groups of Amur Falcons and other species. Wetland and shrub habitats seldom contained occupied nests. If magpie nests were found in shrubs, Amur Falcons (61.5%) most likely occupied them.

Nests were all aggregated, and only a few pairs bred solitarily (Figure 1). Empty nests were mostly distributed along the outer margin of the study area. The average distance between all magpie nests was 439.3 ± 163.2 m, whereas Amur Falcon nest distances averaged 361.6 ± 206.3 m. The distances of Amur Falcons to allospecifics were on average 361.4 ± 191.6 m. Distances to empty nests were larger (410.8 ± 220.9 m).

**Figure 6:**
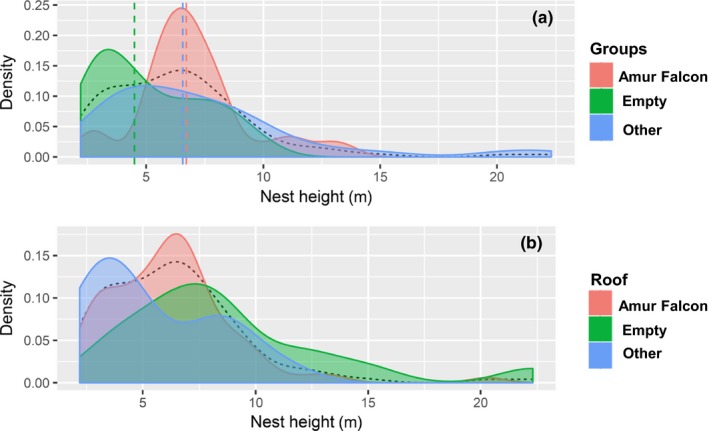
Density plot of the variable nest height for (a) the groups of Amur Falcons, empty nests (empty), and other species (other) and (b) the different categories concerning the status of the roof. The horizontal dashed line shows the average of all groups, and the vertical dashed lines show the medians of the groups

Nests occupied by Amur Falcons were most frequently found at nest heights of about 6.7 ± 1.1 m ranging from 2.5 to 13.5 m (Figure 6). Nests with a domed roof were typically found at around the same height. In almost every third unoccupied nest, a roof was lacking (31.7%). Thus, the group of empty nests comprised the smallest proportions of domed nests (53.6%), 30% less in frequency compared to those occupied by Amur Falcons. The largest variability of nest height was shown by other species starting from a height of 2.3 up to 22.3 m. The smallest nest height was attributed to the Siberian Chipmunk (3.4 ± 0.4 m) in contrast to the Northern Long‐eared Owl (8.7 ± 4.8 m) with the largest one.

Among all groups, Amur Falcons most commonly preferred habitat in their home range which was undisturbed by fire. Only a small proportion of areas frequently disturbed by fire was found within their home ranges (3.4%).

### 3.2 Classification and selection procedure

**Figure 7:**
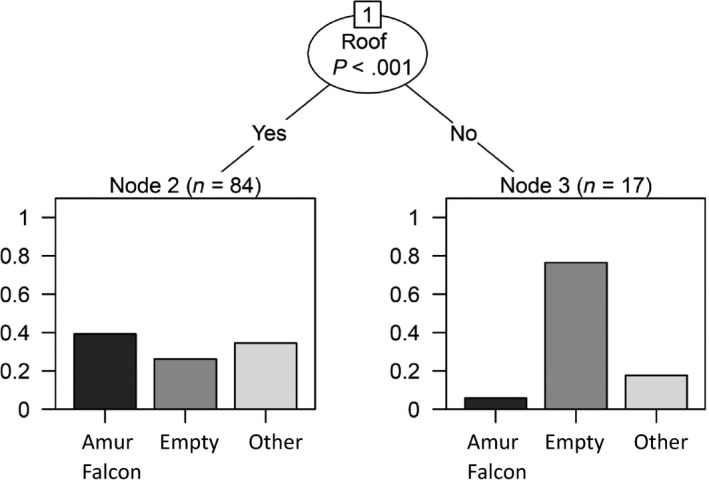
Conditional inference tree for the groups of Amur Falcons, empty nests (empty), and other species (other) classified by the categorical variable roof. Node two accounted for no roof (*n* = 17), whereas node three was attributed to an existing roof (*n* = 84)

Based on the variable *roof*, the sites were split into two distinct groups (*χ* ^2^ *‐test*: *p* < .001): Those equipped with a roof and those without. From the three groups (nests occupied by Amur Falcons, nests occupied by other species and empty nests) used in this analysis, nests without roofs were most often empty and Amur Falcons most often occupied a domed nest (Figure 7).

An “optimal” set of twelve predictor variables remained as serving to predict Amur Falcons' presence with the highest accuracy. The optimal set comprises the following: *roof, total area, mean shape index, splitting index, perimeter–area fractal dimension, proportion of forest patches, proportion of soil patches, proportion of shrub patches, proportion of habitat rarely burned (zero to two times within the last 18 years), nearest wetland patch, nearest empty nest,* and *nearest allospecific nest*.

**Figure 8:**
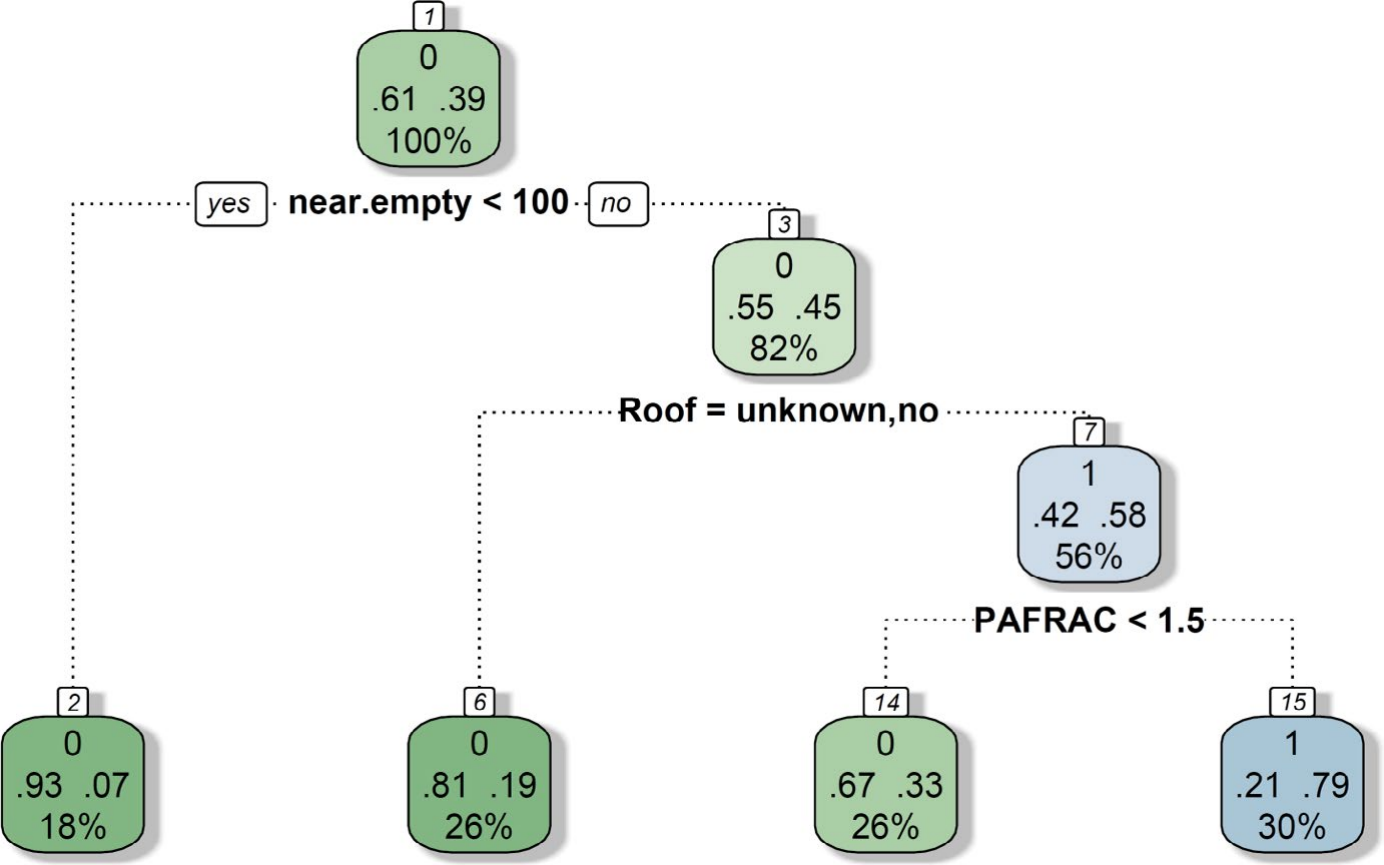
Classification tree structure depicting the variables that best split the dataset into homogeneous subsets to classify the presence of Amur Falcons (1) or their absence (0). Following the right branches, which always display the negation of the stated condition, will lead to the classification of nests occupied by Amur Falcons. Nearest empty nest (near.empty) and perimeter–area fractal dimension (PAFRAC) were given

The variable *nearest empty nest* was able to segregate empty nests and nests occupied by other species than Amur Falcon (group 0) into two homogeneous subsets of the dataset (Figure 8). This was accomplished by a partitioning accounting for distances above and below 100 m. 18.0% of the allospecific and empty nests had shorter distances to the nearest empty nest in reference to the stated condition. We found that the variable *roof* was the best predictor as a second splitting variable in the decision tree model for the presence or absence of Amur Falcons. A proportion of 56.0% can be attributed to nests occupied by Amur Falcons, when the status of the nest was defined as being roofed. As a third predictor variable, the *perimeter–area fractal dimension* separates the remaining data into two groups: Amur Falcons and all other occupied or empty nests (Figure 8).

**Figure 9:**
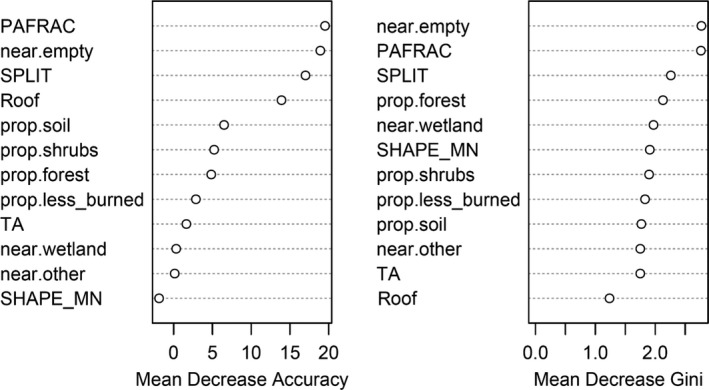
Ranking of the variables used in the random forest application. The larger the values, the more important the variable during the classification procedure. Perimeter–area fractal dimension (PAFRAC), splitting index (SPLIT), mean shape index (SHAPE_MN), total area (TA), roof, nearest wetland patch (near.wetland), empty nest (near.empty) and allospecific nest (near.other), proportion of forest patches (prop.forest), bare soil patches (prop.soil), shrub patches (prop.shrub), and habitat rarely burned (prop.less_burned) were given

Figure 9 shows that the variables *perimeter–area fractal dimension, nearest empty nest,* and *roof* had the most explanatory power within the *random forest* application. If those variables would be left out in the classification process, the mean accuracy of the prediction would decrease significantly as given by the MDA values (Table 1).

**Table 1:** Summary of the predictor variables

	Predictor variable	MDA	*p* (*Χ* ^2^‐test)
Nest characteristic landscape metrics	Roof	13.91	.0142
	PAFRAC	19.53	.0013
	SPLIT	17.01	.06
	TA	1.64	.5237
	SHAPE_MN	−1.88	.2644
Distance relations	Nearest empty nest	18.92	.003
	Nearest wetland	0.33	.8307
	Nearest other nest	0.14	.4548
Proportions	Proportion soil	6.51	.17
	Proportion shrubs	5.22	.1497
	Proportion forest	4.85	.4513
	Proportion less burned	2.87	.437

Note

The Mean Decrease Accuracy (MDA) values from the random forest application and the *p*‐values from the chi‐square test are given for the predictor variables that make up the final variable selection. The set of influential variables comprises the following (from first to last): *roof, perimeter–area fractal dimension, splitting index, total area, mean shape index, nearest empty nest, nearest wetland patch, nearest allospecific nest, proportion of soil, shrubs and forest patches, and proportion of habitat rarely burned (zero to two times within the last 18 years)*. The predictor variables that have a MDA above 10 and a *p*‐value < .05 are highlighted in gray.

*Random forest* showed the greatest overall performance in classifying the nests occupied by Amur Falcons (Table 2). This was confirmed by high AUC values and low error estimates of the model validation.

**Table 2:** Summary of the model validation

Model	ctree (AUC/error)		rf	svm		lg	nnet
Micro							
Full model	0.83/0.23	0.96/0.10			0.80/0.26	0.76/0.29	0.52/0.36
Reduced model	0.69/0.26	0.84/0.21			0.79/0.22	0.74/0.23	0.86/0.17
Macro							
Full model	0.83/0.17	0.98/0.08			0.95/0.06	0.84/0.15	0.50/0.68
Reduced model	0.82/0.15	0.98/0.07			0.95/0.09	0.81/0.27	0.50/0.68
Final red. model	0.76/0.23	0.97/0.06			0.91/0.14	0.81/0.27	0.64/0.42
Optimal model	0.78/0.24	0.97/0.06			0.88/0.17	0.85/0.21	0.50/0.68

Note

The AUC value and the overall error are given for each model and machine learning application; classification tree (ctree), random forest (rf), support vector machine (svm), logistic regression (lg), and neural network (nnet) on a nest‐site scale (micro) and landscape scale (macro). The lowest AIC values were reached for the optimal model, and thus, values from this model are highlighted in gray.

## 4 DISCUSSION AND OUTLOOK

### 4.1 Ecological considerations

Our results suggest a preference of Amur Falcons for a habitat mosaic with many open areas, adequate for foraging, such as wetlands and cultivated fields, and confirm a dependence on magpie nests, favorably equipped with a roof.

As a good explanatory predictor variable, the presence of a roof was indicated to have an influence on the presence of Amur Falcons. A possible explanation is sufficient concealment and protection from predators and adverse weather conditions (Quesada, 2007). The roof might also act as a proxy for nest age—usually, new magpie nests are equipped with a roof (von Blotzheim, 1993). The age of the nests may play a valuable role, since higher ectoparasite loads have been reported for older magpie nests (Zhou et al., 2009). For this reason, most magpies built new nests every year (Antonov & Atanasova, 2003). Amur Falcons might prefer nests which are one to two years old and most probably dominate among other species in the occupation or usurpation of such a nest (Zhou et al., 2009).

The analysis of the nesting habitat and the proportion of forest patches within the home ranges revealed that forest islands within the wetlands commonly served as actual nesting sites. Thereby, they might fulfill a variety of functions such as governing shelter from adverse weather conditions and aerial predation, given the trees have a sufficient canopy. The forest islands display important breeding sites for magpies and obligate nest‐cleptoparasites such as Amur Falcons of whom most of the nests are placed within trees.

Considering the proportion of bare soil within the home ranges, this habitat of mostly fallow land may be supportive for ground‐foraging activities. This has been described for magpies and Red‐footed Falcons (a close relative of the Amur Falcon), especially when soil invertebrates are driven to the surface due to high groundwater levels, providing excellent feeding opportunities (Birkhead, 2010; Palatitz et al., 2015). The importance of agricultural fields might be explained for a similar reason: When the soil is ploughed and during harvest, prey resources might then be exposed for possible predation (Palatitz et al., 2015; Weaver, 2015). Amur Falcon home ranges were also found to be positively associated with shrubs, which might also be associated with prey availability, since habitat heterogeneity might lead to increased insect abundance (Tews et al., 2004).

Furthermore, we found that areas, which burned no more than two times within the last 18 years, were more likely to be found in the home ranges of Amur Falcons, compared to habitats that have been affected by fire more frequently. The greater proportion of habitats burned more frequently within the radius around empty nests can be either attributed to the greater amount of wetlands therein, as wetlands burn more frequent in our study area (Heim et al., 2019), or proof avoidance behavior. Early breeding species like magpies or Northern Long‐eared Owls will most likely abandon their nest during a spring fire, while late‐arriving species (i.e., arrival after the spring fire season) might avoid nests in burned trees, as they might be less concealed due to missing leaves.

Based on the proximity measures on the landscape level, we found that distances to wetlands exerted some influence on predicting the presence of Amur Falcons. Wetland habitat may act as optimal areas for foraging since its high abundance of insects and the lack of vertical structures, providing good flight conditions (Brazil, 2009). Red‐footed Falcons have been observed to forage commonly above wetlands (Bertau, 2014). However, a great amount of wetland patches encompasses the area particularly around empty nests.

The results from the random forest model indicate that the distance to nests occupied by other species and empty nests are significant predictors. This is an interesting result, since medial distances from Amur Falcon nests to conspecific and allospecific nests are almost identical, whereas the median of the distance to empty nests is indicated to be the highest for nests occupied by Amur Falcons. However, the range of distances among each magpie nest shows great variability. The median of the magpie nest distances matches to values from the literature (Birkhead, 2010; Zhou et al., 2009). The review of many studies by Birkhead (2010) shows that distances between the nearest magpie nests average 242.4 m. The study of Zhou et al. (2009) presents that distances between magpie nests occupied by raptors have greater values (151.1 m) in comparison with distances between empty nests and raptor nests (67.6 m). The results of our study reveal a similar pattern, although the differences are rather small. The distance of occupied nests (whether by Amur Falcons or other species) to empty nests is on average slightly smaller (396.1 m) than between occupied nests (409.9 m) in our study area. The average distance between Amur Falcon nests is given as 201.5 m in the study of Schaefer (2003) ranging from 3.0 to 766.0 m. Our study indicates an average distance of 361.6 m between nests of Amur Falcons with particular nests exceeding a distance of 766.0 m. These findings suggest that the magpie nests at our study site are more spread out in respect to the aforementioned studies. Magpie nest distances can have a great range, suggesting that the species can cope and also benefit from being in close associations to each other (Baeyens, 1981; Birkhead, 2010). However, most of the nests are spaced out in a way that, within the immediate vicinity around a nest, no direct interaction would occur. Nonetheless, overlapping home ranges allow encounters between allospecifics and conspecifics.

The necessity to cope with breeding neighbors in close aggregations is also relevant for Amur Falcons. In the early stages of the breeding season, there is an increased risk of predation to the hatched young, regarding both Amur Falcons and magpies (Schaefer, 2003). For this reason, it can be beneficial to keep a certain distance and prevent possible attacks.

The results of the study relate to other studies where landscape structure, represented by various metrics, had an effect on species' selection of the breeding habitat (Barbaro & Van Halder, 2009; Berry, Bock, & Haire, 1998; Bomhard, 2002; Jokimäki & Solonen, 2011; Jones, 2001; Massey, Bowen, Griffin, & McGarigal, 2008; Wiens, Chr, Horne, & Ims, 1993). Differences in the total area of the presumed home ranges of all magpie nests were likely caused by the arbitrarily chosen borders of the study area. Thus, the nests at the outer margin did not cover the full home ranges compared to nests in the center of the study area. A structurally complex landscape, indicated by high splitting index values in our model, might be a relevant feature for a species, if it depends on a variety of prey species during the course of a year. A more complex and diverse structure in the shape of the habitats, as indicated by relatively large perimeter–area fractal dimension values in our model for the Amur Falcon, means an increase of edge zones within the landscape. It could also refer to an increase in the number of gradients from patch to patch along with changes in biotic and abiotic factors such as vegetation characteristics and humidity. Nevertheless, magpies and Amur Falcon might regularly take advantage of edge habitats, because insects' abundance and detectability might be especially high in those ecotones (Birkhead, 2010; Palatitz et al., 2015).

As an open wetland species, Amur Falcons are well‐adapted to catch insects in flight and therefore depend on a certain amount of open areas with high insect abundance for foraging (Brazil, 2009; Ristow, 2004). A suitable habitat composition for this raptor can be confirmed by our study, since forested patches only comprise areas of about 17% on average. Amur Falcons most likely make temporary use of different habitats regarding seasonal variations in food demands and abundances (Kopij, 2010; Purger, 1998; Ristow, 2004; Symes & Woodborne, 2010). Consequently, the overall habitat use and foraging strategies might change in relation to changes in prey abundance and detectability, which is influenced by the actual vegetation cover in proceeding stages of the growing season (Palatitz et al., 2015). Above all, Amur Falcons may offset a less optimal set of habitat arrangements by their gregarious behavior, flock formation, and their ability to fly over a distance, where abundance and particularly detectability of prey items are sufficient (Palatitz et al., 2015). However, our study assumes that variables at the landscape scale, such as those referring to structurally complex landscapes with proportions of wetlands, shrubs, and cultivated fields, helped to classify nests of Amur Falcons.

A key assumption behind the whole discussion is to expect that Amur Falcons actively choose their habitat and nesting sites. One has to emphasize that the selection process described is a multilevel process. The occupation of magpie nests by Amur Falcons underlies the selection procedure of magpies. The magpies in turn strongly depend on available nest sites, such as suitable trees, and sufficient food resources within the landscape (Birkhead, 2010; Zhou et al., 2009). Thus, the remaining forest and wetland patches in the study area are of considerable value for magpies, and all species depending on their nests, including the Amur Falcon. “Nest‐cleptoparasitism” could become a disadvantage, if the location of proper nests does not coincide with suitable foraging habitats.

Nest sites provided by magpies are used by a good amount of other species and possibly cause interspecific reactions such as competition among bird communities (Prokop, 2004; Zhou et al., 2009). The composition and configuration of probable food habitats may be relevant for the Amur Falcon, but it remains difficult to prove to which degree habitat selection was performed due to structural elements of the landscape, especially, when considering the intra‐ and interspecific competition and the significant dependency of Amur Falcons on the availability of magpie nests. However, the constitution and distribution of habitats might play a crucial role in determining suitable nesting grounds (Newton, 1979). A trade‐off can be expected between the species need and the available resources concerning food, shelter, and lookout (Baeyens, 1981; Charman, Smith, Dodd, Gruar, & Dillon, 2012; Lipsey, Naugle, Nowak, & Lukacs, 2017; Møller, 1991; Riffell et al., 2015; Stout, Temple, & Cary, 2006; Vrezec & Tome, 2004).

The final paragraph discusses the benefits and shortcomings of the statistical tools used in this study. Generalized linear models and other traditional statistical methods are seen to be inadequate to reveal patterns and relationships of interdependent variables that can be uncovered by more novel procedures from the field of data mining (Cutler et al., 2007). McGarigal and McComb (1995) suggest applying different analytical approaches to gain thorough insights into the data and to avoid limitations of a single statistic. Hochachka et al. (2007) recommend methods of data mining, such as random forest and decision trees, to analyze ecological data in order to extract as much information as possible from the available data. Random forest has the power to analyze data that include nonlinear and complex interactions among predictors (Cutler et al., 2007). Nevertheless, it remains difficult to understand the rules that lead to model outcomes, and in order to classify a new dataset, the entire forest needs to be stored (Kabacoff, 2015). Therefore, ecological interpretations are impeded since simple representations are not available for the random forest application, such as the pictorial graphs of decision trees. However, random forest is described as being competitive or even superior to the most common statistical methods and serves as an effective tool to detect patterns within the data and allow deriving first ecological hypotheses (Cutler et al., 2007). Finally, every statistical outcome relies on the quality of data, and only with more and long‐term data about relevant variables regarding the nest site and breeding habitat of the Amur Falcon, ecological relationships can be manifested.

In conclusion, we found that Amur Falcons can make use of magpie nests in very different locations, but significantly prefer nests with an intact roof. In addition to the nest‐site selection, our model results indicate that landscape variables around the nest location influence the breeding habitat selection of the Amur Falcon.

## 5 CONCLUSIONS

All nests used by Amur Falcons were built by Eurasian Magpies, confirming its status as a “nest‐cleptoparasite”.

From the pool of available magpie nests, those with a roof (i.e., newly built), situated at a height of about 6–7 m in rarely burned areas, with adjacent patches of shrub, forest, cultivated field, and wetland habitat, and a nest location with a distance of around 360 m to allospecific nests are the ones most likely usurped by Amur Falcons in our study area.

The machine learning algorithm random forest most precisely selected influential variables to predict the Amur Falcon occurrence probability, providing the highest accuracy among the tested classification methods.

## CONFLICT OF INTEREST

The authors confirm that there are no competing interests.

## AUTHOR CONTRIBUTIONS

MF has planned and conducted data analysis. WH has conceived the study. AH has prepared the habitat classification map. AH, FM, MB, SMS, and WH took part in the field work and data collection. R‐UM helped to supervise data analysis and manuscript preparation. MF and WH have written the manuscript. All authors have commented on the manuscript.

## Data Availability

The magpie nest data are available on Dryad (https://doi.org/10.5061/dryad.s4mw6m93d). All other data used in this study are available from the publications cited.
